# The New Housing Policy

**Published:** 1923-04

**Authors:** 


THE
### HOSPITAL and HEALTH
REVIEW

### THE NEW HOUSING POLICY.

With the warnings of three adverse by-elections ringing in his ears, Mr. Neville Chamberlain, the new Minister of Health, has lost no time in giving the country an outline of the essential proposals of the Government for removing, or mitigating, the housing crisis. Those elections were lost, in the main, because the Cabinet seemed unable to make up its corporate mind on the one domestic subject which was exciting the electors and because its proposals in so far as they were made known, alarmed a large section of them. Since it is obviously necessary, however regrettable it may be, to continue restriction for some time to come, the policy Mr. Chamberlain has announced is probably the cheapest and most practical that could be devised, especially if, as there is good ground to hope, it is reinforced by valuable collateral provisions. In principle the Government have accepted the "Manchester Programme." An annual subsidy of £6 per house is to be granted for twenty years for five-roomed houses of a "non-parlour" type. It is believed that this sum will cover one-half of the annual loss, the other half being made good from the rates. The great northern municipalities, with whom the proposal originated, asked that the subsidy should continue for sixty years, but that was obviously excessive, since it would have meant that by the end of the period the Treasury and the local authority would have lost, between them, £720 on each house, which is very nearly double what, in many localities, these dwellings will cost. It is understood that the rent of this smallest type of artisans' homes is to be seven shillings a week.

If this scheme were confined to the Councils we should expect it to go a very short way towards solving a problem which has caused, and is still causing, a vast amount of discomfort and no little unrest. Happily the subsidy is to be available also to private enterprise—which means, in effect, the speculating builder and those who finance him. In years gone by there was a well-founded suspicion of this type of builder, but during the past generation building by-laws have been so greatly strengthened, and they are now, as a rule, so firmly enforced, that there is reason for gratitude to, rather than suspicion of, the man who, before the war, provided nine-tenths of the houses built in this country. It is true that, the subsidy being confined to the smallest type of house, the serious needs of the lower middle-class will not be directly ministered to. For help in that direction—and it is help that is sorely needed—we must look to the adoption by the Government of some of the collateral plans to which we have alluded. No single cut-and-dried scheme will ever relieve us of our difficulties. It is essential that there should be a variety of schemes working simultaneously, directed to the provision of differing classes of houses. If, for example, the Government would guarantee the building societies against loss, on condition that they advanced 90 per cent. of the cost of building, we cannot doubt that many small capitalists would be eager to provide their own roof-tree. If only new houses of the smaller kind could be exempted from rates for a limited term of years building would receive an impetus which could be imparted by no other device. In New York such exemption has been successful beyond all expectation.

The details of the Government plan have yet to be filled in, and until they are known it is impossible to pronounce a verdict upon their policy as a whole. It is obvious that precautions will have to be taken to insure that the local authorities really will give proper facilities to local enterprise, since in the new circumstances they will be placed in the position the

Ministry of Health occupied under the unhappy Addison scheme. A grave responsibility will rest upon them in this respect. An even greater responsibility rests upon the Government to make good their promises, at present rather shadowy, to give to private individuals the same powers for the clearance of slums that have long been possessed by the municipal authorities. The abolition of slums must proceed side by side with the supply of houses—the two subjects are closely intertwined. So long as there is an insufficiency of houses the creation of slums will continue. We trust that these considerations will be kept well in the forefront of the discussions upon the coming legislation. We cannot afford to lose the present opportunity of dealing with a matter of vital importance to the physical and moral health of the people. It is impossible to rear a robust, self-reliant, sane and physically fit generation in a country where decent dwellings are difficult to obtain and where slums make their endless contribution to the ranks of the vicious and the diseased.

